# Advanced Fuzzy Potential Field Method for Mobile Robot Obstacle Avoidance

**DOI:** 10.1155/2016/6047906

**Published:** 2016-03-30

**Authors:** Jong-Wook Park, Hwan-Joo Kwak, Young-Chang Kang, Dong W. Kim

**Affiliations:** ^1^Department of Electronic Engineering, Incheon National University, Incheon 402-752, Republic of Korea; ^2^Powertrain Platform Team, Hyundai Autron Co., Ltd., Mtek IT Tower, No. 344, Pangyo-ro, Bundang-gu, Seongnam-si, Gyeonggi-do 463-400, Republic of Korea; ^3^Department of Computer Engineering, Gachon University, 1342 Seongnamdaero, Sujeong-gu, Seongnam-si, Gyeonggi-do 461-701, Republic of Korea; ^4^Department of Digital Electronics, Inha Technical College, 100 Inha-ro, Nam-gu, Incheon 402-752, Republic of Korea

## Abstract

An advanced fuzzy potential field method for mobile robot obstacle avoidance is proposed. The potential field method primarily deals with the repulsive forces surrounding obstacles, while fuzzy control logic focuses on fuzzy rules that handle linguistic variables and describe the knowledge of experts. The design of a fuzzy controller—advanced fuzzy potential field method (AFPFM)—that models and enhances the conventional potential field method is proposed and discussed. This study also examines the rule-explosion problem of conventional fuzzy logic and assesses the performance of our proposed AFPFM through simulations carried out using a mobile robot.

## 1. Introduction

The subject of mobile robots has continuously been one of the most important and attractive subjects in the field of robotics. The efficient utilization of a robot is predicated on its mobility. As a result, many researchers have investigated ways to improve this feature [1–5]. This paper primarily concerns the obstacle avoidance capabilities of a mobile robot. Obstacle avoidance is one of the most important abilities for the safe and reliable movement of a mobile robot. The potential field method (PFM) is the most favored and widespread online obstacle avoidance method for mobile robots because of its computational simplicity and efficiency [6–10]. The concept of an artificial potential field was applied to obstacle avoidance by Khatib in 1985 [11]. His algorithm evolved from the intuitive idea that a robot is attracted to a goal and repulsed by obstacles. By harnessing these repulsive and attractive forces, a robot can avoid colliding with surrounding obstacles and reach its goal safely. However, despite all its advantages, the PFM was abandoned due to limitations such as the lack of a passage between closely placed obstacles and oscillations in narrow passages [12]. The vector field histogram (VFH) was introduced by Borenstein and Koren as an alternative obstacle avoidance method [13–15]. Ulrich and Borenstein improved the VFH and extended it to VFH+ [16] and VFH*∗* [17]. The VFH technique generates a polar histogram from which all openings large enough to pass through are identified. Methods based on the VFH can select a central path through a passage identified by the polar histogram and eliminate the fluctuations in steering control with the averaging effect of the polar histogram. However, VFH techniques have some drawbacks. They all use a two-dimensional histogram grid as a world model, which they update continuously with data received from range sensors. To process this histogram, VFH techniques may require more storage space and more computational cost than the PFM. Therefore, these techniques are not appropriate for robot systems that have only small computational power and that should allocate storage space and computation time as small as possible to obstacle avoidance. In addition, in VFH techniques, the accuracy of the performance depends on how correct the accumulated information of the histogram is. If the histogram is damaged by unpredicted disturbance, the robot might fail to identify the correct openings and consequently may not be able to maneuver through passages. Furthermore, in VFH techniques, performance is extremely unstable and sensitive to the threshold value [18]. If the threshold is too low, the robot cannot pass through narrow passages; if it is too high, the robot may collide with obstacles. Using the threshold, VFH techniques quantize the high-level certainty values of the histogram into only two levels and abandon the beneficial information that can be derived from surrounding obstacles. Because only information on the candidate direction (selected by the threshold) is given, the robot cannot perform efficient and stable obstacle avoidance. Finally, as is the case with the PFM, obstacle avoidance using VFH techniques cannot overcome the local minima problem. Both obstacle avoidance methods (the PFM and VFH techniques) are local path planners, and they therefore need the additional escape techniques available to a global path planner in the local minima situation. Although the VFH techniques were proposed with the aim of overcoming the drawbacks of the PFM, they also have some critical flaws. Therefore, this study proposes overcoming the limitations of both the PFM and the VFH techniques by presenting a new obstacle avoidance method for mobile robots using an advanced fuzzy PFM (AFPFM). The proposed obstacle avoidance method based on the PFM driven by fuzzy logic improves on all the advantages of the PFM [19, 20]. The simple and intuitive characteristics of the AFPFM (inherited from the PFM) enable easy implementation and low computational cost. In addition, using recent sensor values frees the robot from the sort of problems that occur when using incorrect information accumulated by the VFH. Therefore, our proposed method can be widely applied even to robots that suffer from a lack of computational capacity, unpredictable disturbance, and kidnapping.

## 2. Advanced Fuzzy Potential Field Method: AFPFM

### 2.1. Model of Mobile Robot and Conventional PFM

**Figure 1:**
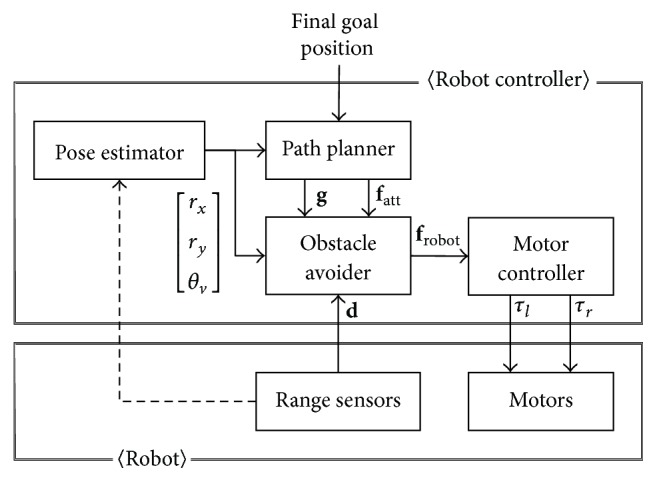
The two sections of the robot system.

The considered robot system in this paper has two main parts: the robot controller and the robot, as illustrated in Figure 1. The robot controller consists of the position and orientation (POS) estimator, path planner, obstacle avoider, and motor controller. At a minimum, the robot consists of motors and range sensors. The robot and its controller are configured with feedback. The robot controller controls the motors of the robot and can change its POS and velocity. When the robot moves, the measured values from the sensors are fed back to the robot controller. In typical mobile robots, the POS estimator and obstacle avoider reference information about surrounding obstacles (provided by the attached sensors) to perform localization and obstacle avoidance, respectively. In this paper, however, we assume that the odometry of the robot is ideal and therefore do not consider the connection from the range sensors to the POS estimator.

Based on the POS of the robot and the goal position, the path planner finds a path for the navigation of the mobile robot from the start position to the goal position. After that, the robot tracks the planned path to reach the goal. Most mobile robots, however, operate in nonstationary environments consisting of changing surroundings and do not have complete information about the surrounding environments even in familiar environments. To avoid a collision with unknown surroundings, the obstacle avoider modifies the path given by the path planner, after referencing the POS parameters [*r*_*x*_ *r*_*y*_ *θ*_*v*_]^*T*^ given by the POS estimator. In this paper, we propose a new design method for the obstacle avoider.

**Figure 2:**
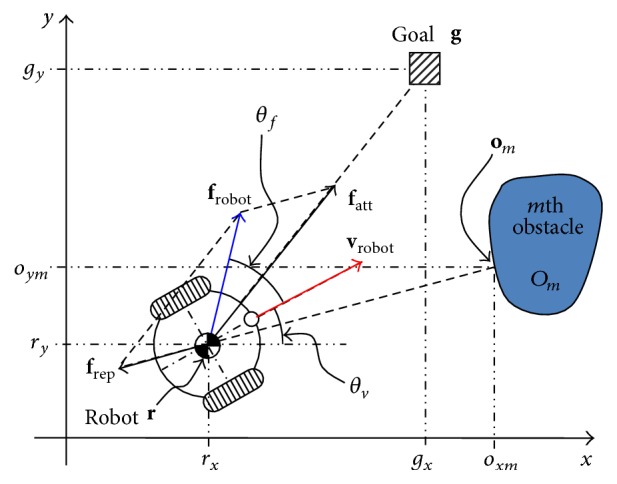
Coordinate system and control value.

Figure 2 describes the coordinate system and control values of the considered mobile robot. The positions of the mobile robot, *m*th obstacle, and goal are denoted by (1), (2), and (3), respectively. As with most robot systems, we also apply a discrete system (with sampling period *t*_*s*_) as a controller:

(1) rtk=rxtkrytkT,

(2) qi=qixqiyT,

(3) g=gxgyT,

where *t*_*k*_( = *kt*_*s*_) is the *k*th sampling time, and the heading direction of the robot at *t*_*k*_ is denoted by *θ*_*v*_(*t*_*k*_). If the robot is equipped with *n* range sensors, the measured distances of these sensors and the heading direction of the *i*th sensor are described as follows:

(4) dtk=d1tk⋯dntkT,sitk=cos⁡θvtk−sin⁡θvtksin⁡θvtkcos⁡θvtksi0,

where **s**_*i*_(0) is the initial heading unit vector of the *i*th range sensor.

In the potential field method, the motion of the robot is constrained to follow the direction of the artificial force **f**_robot_ that can be rewritten as

(5) $$\begin{array}{c} f_{robot}=f_{att}+f_{rep}, \end{array}$$

where **f**_att_ is the attractive force of the goal and **f**_rep_ is the repulsive force of the obstacle. The practical artificial force **f**_robot_ that controls the robot is a combination of the attractive and repulsive forces. The attractive force can be defined as

(6) $$\begin{array}{c} f_{att}=\left\{\begin{array}{cc} k_{att}\left(g-r\right), & \text{if }\left\|g-r\right\|<d_{goal},\\ \frac{d_{goal}k_{att}\left(g-r\right)}{\left\|g-r\right\|}, & \text{otherwise,} \end{array}\right. \end{array}$$

where *k*_att_ is the positive constant of the attractive forces and *d*_goal_ is the threshold distance of the goal. If the robot is in the boundary *d*_goal_ of the goal, the attractive force **f**_att_ is the gradient of the conic potential, *k*_att_(**g** − **r**). Furthermore, the repulsive force **f**_rep_ between the robot and the obstacles prevents a collision and can be induced by the sum of the negative gradients of the potential field, as in

(7) frep=krep∑i=1n1r−oi−1dmaxr−oir−oi,if  r−oi<dmax,0,otherwise,

where *n* is the number of surrounding obstacles, *k*_rep_ is the positive constant of the repulsive forces, and *d*_max_ is the influence distance of the obstacles. The closest point **o**_*i*_ of the *i*th obstacle *O*_*i*_ is defined as

(8) $$\begin{array}{c} o_{i}=\underset{c_{i}\in O_{i}}{\arg\;\min}\left\|\;r-c_{i}\;\right\|, \end{array}$$

where *O*_*i*_ means all points in the *i*th individual convex obstacle.

**Figure 3:**
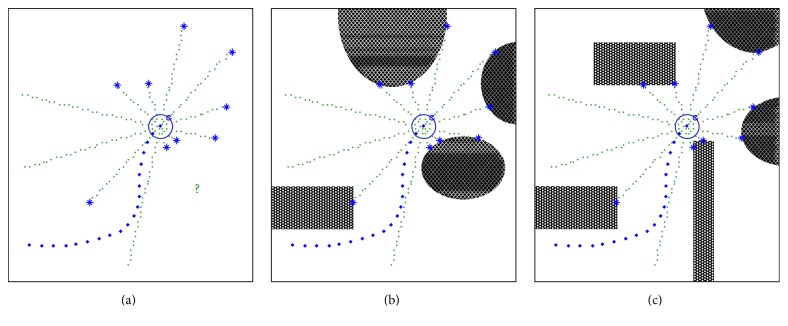
Obstacle recognition problem in unknown environments.

If the PFM employs only the repulsive force of the closest obstacle in numerical implementation, the robot may oscillate at the points where it is between multiple obstacles. Therefore, the repulsive force **f**_rep_ of the practical PFM is a combination of *n* subrepulsive forces of *n* individual obstacles, instead of the repulsive force of the closest obstacle. For the practical implementation of the PFM, the closest point **o**_*i*_ of the *i*th obstacle *O*_*i*_ should be recognizable. The robot, however, only knows the distances from itself to the obstacles, which are measured by the sensors equipped in their respective directions. If the result of the distance measurement is as shown in Figure 3(a), then the robot will not be able to determine the correct state of the surrounding obstacles with only this poor result. The actual surrounding obstacles may be expected to be in various shapes and various positions, as illustrated in Figures 3(b) and 3(c).

### 2.2. Concept of Obstacle Approximation

**Figure 4:**
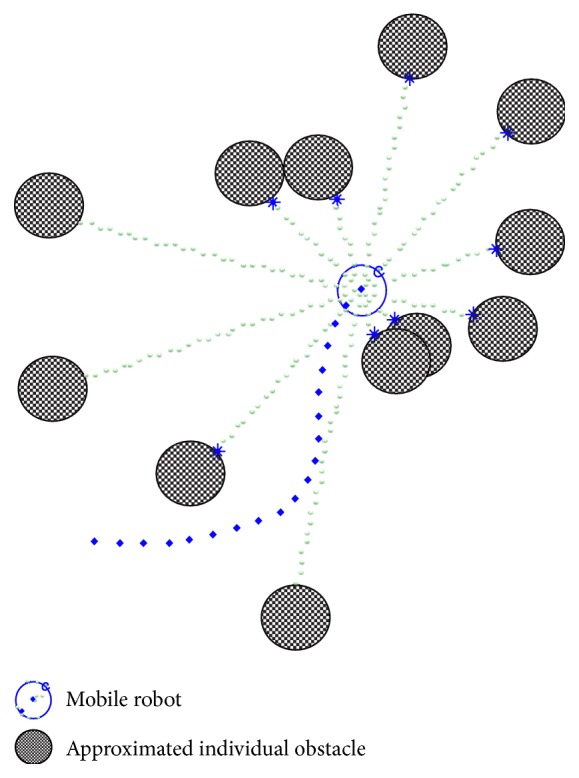
The surrounding obstacles, approximated by convex particles.

To deal with the obstacle recognition drawback in the practical implementation of the PFM, we assume that multiple small convex particles are placed in the direction of their respective sensors and all these particles are considered to be individual obstacles, as depicted in Figure 4. In accordance with this approximation, the heading direction vector of the *i*th sensor **s**_*i*_ can be written as

(9) $$\begin{array}{c} s_{i}=-\frac{r-o_{i}}{\left\|r-o_{i}\right\|} \end{array}$$

and the repulsive force **f**_rep_ can be rewritten as

(10) $$\begin{array}{c} f_{rep}=\left\{\begin{array}{cc} -k_{rep}\overset{n}{\underset{i=1}{\sum }}\left(\frac{1}{d_{i}}-\frac{1}{d_{max}}\right)s_{i}, & \text{if }d_{i}<d_{max},\\ 0, & \text{otherwise,} \end{array}\right. \end{array}$$

where *d*_*i*_ is the distance measured by the *i*th sensor.

### 2.3. Design of Advanced Fuzzy Potential Field Method

The repulsive force, which is the main concept of the PFM, is a combination of the respective subrepulsive forces of all the individual surrounding obstacles, as defined by (7). Similarly, the output of the fuzzy controller is a combination of several fuzzy rules. Assume that one fuzzy rule of the fuzzy controller can describe the corresponding subrepulsive force of one surrounding obstacle, and the combination of *n* fuzzy rules can describe the sum of all subrepulsive forces of *n* individual obstacles. If the robot is surrounded by *n* individual obstacles and the controller of this robot is a fuzzy controller with *n* fuzzy rules, then this fuzzy controller can fully model the performance of the conventional PFM. Consequently, the controller design for the fuzzy PFM (FPFM), which substitutes for the conventional PFM using the fuzzy controller, should consider three factors:

1. The number of fuzzy rules is the same as the number of subrepulsive forces.
2. One fuzzy rule can describe the corresponding subrepulsive force of one obstacle.
3. The combination of all fuzzy rules is a numerical sum of all the subrepulsive forces of the obstacles.

The first factor deals with the concept of obstacle approximation stated before. The most practical robot with low-resolution range sensors cannot determine the actual number of individual obstacles. Therefore, we assume that each sensor detects its respective obstacle, and the number of individual surrounding obstacles is the same as the number of sensors. Because the number of subrepulsive forces is the same as the number of individual surrounding obstacles in the conventional PFM, the number of fuzzy rules in the FPFM can be determined to be the same as the number of sensors.

Second, the subrepulsive forces of the conventional PFM can be imitated using the fuzzy rules designed below. The fuzzy controller has *n* scalar inputs (i.e., *d*_1_,…, *d*_*n*_), one vector output **y**, and *n* fuzzy rules. It uses the Takagi-Sugeno fuzzy model [21], which is described by a set of “IF-THEN” rules as follows:

(11)  pth  Rule  Rp: IF  dp  is  μdp THEN  yp=∑i=1nε+didmaxdmaxε+dpkrep1dp−1dmaxsp,

where *p* = 1,…, *n* with *n* being the total number of all fuzzy rules, *ε* > 0, *μ*_*d*_*p*__ is the membership function for input *d*_*p*_, and **s**_*p*_ is the heading direction vector of the *p*th sensor.

Finally, the defuzzification equation of the controller is the weighted average of all the outputs, as follows:

(12) $$\begin{array}{c} y=\frac{\sum_{p=1}^{n}w_{p}y_{p}}{\sum_{p=1}^{n}w_{p}}, \end{array}$$

where the *p*th weight value is *w*_*p*_ = *μ*_*d*_*p*__(*d*_*p*_). For the equivalent repulsive force of the conventional PFM and the fuzzy control system, a proper membership function *μ*_*d*_*p*__ for the *p*th fuzzy rule must be designed based on the designed fuzzy rules, and the defuzzified overall output of the fuzzy controller in (12) must be equivalent to that of the PFM. From all *n* outputs of (11) and the defuzzification equation (12), the output of the fuzzy controller is

(13) y=krep∑i=1nε+didmax·∑p=1nwpdmax/ε+dp1/dp−1/dmaxsp∑p=1nwp.

If the *p*th weight value (i.e., membership function *μ*_*d*_*p*__ for the *p*th fuzzy rule) is designed as

(14) $$\begin{array}{c} w_{p}=\mu_{d_{p}}\left(\;d_{p}\;\right)=\frac{\varepsilon +d_{p}}{d_{max}} \end{array}$$

and substituted into (13), then the output of the fuzzy controller will be identical to that of the conventional PFM. The outputs of the fuzzy rules in (11) are weighted by (14) and averaged by (12). As a result, the overall output of the fuzzy controller is the same as that in (10), and each output of the *p*th fuzzy rule represents the repulsive force of the *p*th obstacle, which is described as the potential function. As this parameter of the fuzzy rule increases, the repulsive force gets powerful and the obstacle pushes the robot strongly.

From (10), the repulsive force of the conventional PFM can be rewritten as follows:

(15) $$\begin{array}{c} f_{rep}=-\overset{n}{\underset{p=1}{\sum }}f_{PFM}\left(\;d_{p}\;\right), \end{array}$$

where

(16) $$\begin{array}{c} f_{PFM}\left(\;d_{p}\;\right)=\left\{\begin{array}{cc} k_{rep}\left(\frac{1}{d_{p}}-\frac{1}{d_{max}}\right)s_{p}, & \text{if }d_{p}<d_{max},\\ 0, & \text{otherwise.} \end{array}\right. \end{array}$$

The repulsive force is the sum of *n* subrepulsive forces, and the magnitude of the subrepulsive forces is only with respect to the distances between the robot and the obstacles in the conventional PFM. Furthermore, the repulsive force of the FPFM, which imitates this conventional PFM, can be rewritten as follows:

(17) $$\begin{array}{c} f_{rep}=-\frac{\sum_{p=1}^{n}w_{p}f_{FPFM}\left(d_{p}\right)}{\sum_{p=1}^{n}w_{p}}, \end{array}$$

where

**Figure 5:**
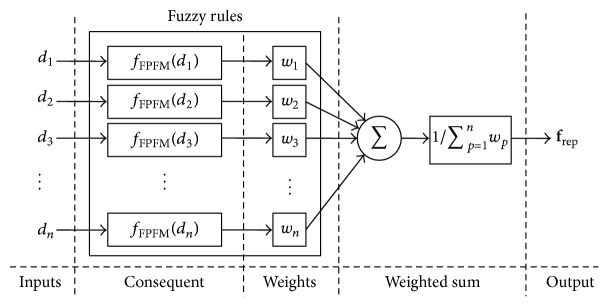
Block diagram of the fuzzy controller for the conventional PFM.

(18) fFPFMdp=∑i=1nε+didmaxdmaxε+dpkrep1dp−1dmaxsp

and the block diagram of the FPFM is as depicted in Figure 5.

The design of the AFPFM can be considered in two steps from FPFM: adding additional fuzzy rules and adding additional control inputs.

The first way in which to improve the FPFM is to add fuzzy rules. The fuzzy “IF-THEN” rule is the basic unit for the linguistic representation of knowledge in fuzzy control systems. Each fuzzy rule represents a part of the control strategies of experts, and a complete collection of them represents the entire control strategies of the experts. The fuzzy “IF-THEN” rule is generally expressed as follows:

 IF (antecedent), THEN (consequent).

The antecedent describes a condition, and the consequent describes a conclusion. The first step to improving the FPFM is to improve the antecedents and consequents of the fuzzy rules.

As shown in Figure 5, the fuzzy control system of the FPFM is a special case of the usual fuzzy control logic, described as follows:

(1) Each antecedent of a fuzzy rule is with respect to only one input variable, *d*_*p*_.
(2) Each input variable is fuzzified by only one membership function, *μ*_*d*_*p*__(·).
(3) All antecedents of the fuzzy rules are identical, that is, *f*_FPFM_(*d*_*p*_).

The antecedent can be improved by adopting complex and complete fuzzy rules, while the consequent can be improved by varying the description of the conclusion. However, these improvements also cause the most challenging problem in the fuzzy control system, that is, the rule-explosion problem [22, 23]. To deal with this problem, in this study, we introduce the design of the distributed fuzzy control system.

For the completeness of the fuzzy rules, all fuzzy rules should cover all the combinational space of the fuzzy inputs. The fuzzy rules of the FPFM, however, do not satisfy the condition of fuzzy rule completeness. From (11), the antecedent of the fuzzy rule is with respect to only one input. In addition, the antecedent of the fuzzy rules of the FPFM has only one membership function, as denoted by (14). To sufficiently describe the control strategies, the antecedent of the fuzzy rules needs to be improved. As a result, the FPFM can be improved by improving and completing the antecedent of the fuzzy rules. The antecedent of the fuzzy rules should consider all input variables from *n* sensors and increase the number of membership functions to *m* > 1. The advanced fuzzy rules can be written as

(19)  p,q1,…,qnth  Rule  Rp,q1,…,qn: IF  d1  is  μd1,q1,…,dp  is  μdp,qp,…,dn  is  μdn,qn THEN  yp,q1,…,qn=fAFPFMdp,

where *p* = 1,…, *n* and *q*_*k*_ = 1,…, *m* for *k* = 1,…, *n*. Furthermore, the repulsive force ${\tilde{f}}_{rep}$> of these advanced fuzzy rules can be written as follows:

(20) $$\begin{array}{c} {\tilde{f}}_{rep}=-\frac{\sum_{p=1}^{n}\sum_{q_{1}=1}^{m}\cdots \sum_{q_{n}=1}^{m}w_{p,q_{1},\ldots ,q_{n}}f_{AFPFM}\left(d_{p}\right)}{\sum_{p=1}^{n}\sum_{q_{1}=1}^{m}\cdots \sum_{q_{n}=1}^{m}w_{p,q_{1},\ldots ,q_{n}}}, \end{array}$$

where

(21) fAFPFMd1,…,dn=∑i=1nε+didmaxdmaxε+dpkrep1dp−1dmaxsp,wp,q1,…,qn=μd1,q1d1∧⋯∧μdp,qpdp∧⋯∧μdn,qndn.

Compared to (11) of the FPFM, the antecedent of the advanced fuzzy rules of (19) has *n* more fuzzy conditions for the added *n* input variables *d*_*q*_1__,…, *d*_*q*_*n*__. The FPFM represents one subrepulsive force with only one fuzzy rule, but the AFPFM with the advanced fuzzy rules of (19) can represent individual subrepulsive forces using multiple fuzzy rules that can describe all the control conditions of the robot.

In addition to this improvement of the antecedent of the fuzzy rules, the FPFM can be enhanced even more by improving the consequent of the fuzzy rules. The above improvement of the antecedent increases the number of fuzzy rules and diversifies the robot's control conditions. These varied conditions must be controlled fully by various and elaborate consequents of the fuzzy rules. The output function *f*_AFPFM_(*d*_*p*_) of each fuzzy rule can be replaced by several nonlinear functions *f*_AFPFM_*p*,*q*_1_,…,*q*_*n*___(*d*_1_,…, *d*_*n*_).

**Figure 6:**
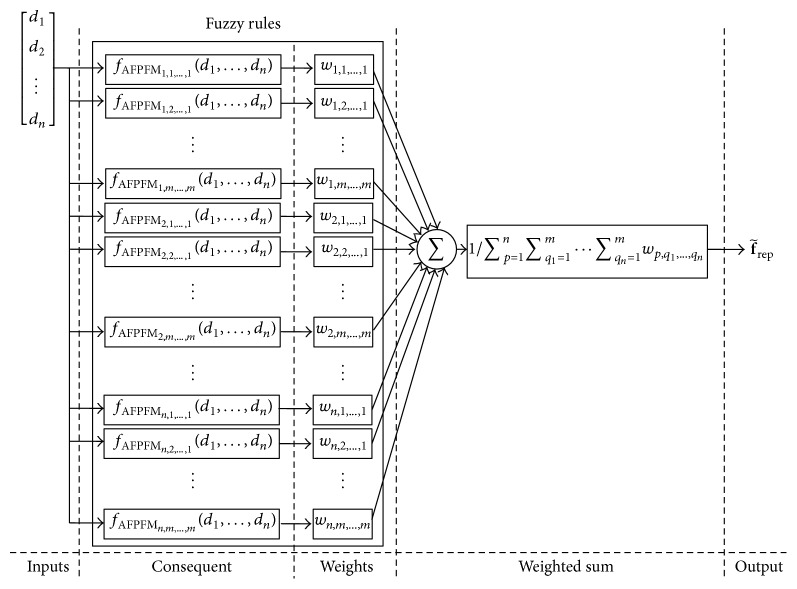
Block diagram of the advanced fuzzy controller created by adding additional fuzzy rules.

A block diagram of the advanced fuzzy controller, made by adding additional fuzzy rules, is shown in Figure 6. Based on the improvement of the antecedent and consequent of the fuzzy rules, this fuzzy controller can fully describe the complex and elaborate control strategies of experts. This advanced fuzzy controller, however, faces the rule-explosion limitation. Compared to the FPFM fuzzy controller, which considers only one input variable and one membership function for each fuzzy rule, the fuzzy rules of the advanced FPFM are with respect to all input variables from the sensors (i.e., *d*_1_,…, *d*_*n*_) and multiple membership functions. Assume that there is a mobile robot equipped with *n* sonar sensors and modeled as in Figure 2. If the fuzzy controller has *m* membership functions for each input variable, then the advanced FPFM demands *n* × *m* ^*n*^ fuzzy rules for the complete construction shown in Figure 6. The larger number of input variables and membership functions increases the number of fuzzy rules exponentially (i.e., the fuzzy rule-explosion problem). To overcome the rule-explosion problem, some researchers have considered the design of a hierarchical fuzzy controller, which dramatically reduces the number of fuzzy rules involved [24, 25]. This controller, however, has other drawbacks such as difficulty of system identification and loss of linguistic interpretability. Building a hierarchical fuzzy controller is more difficult than building a single-layer fuzzy controller. This study proposes the design of a distributed fuzzy controller that combines the advantages of single-layer and hierarchical fuzzy controllers and utilizes it in the design of the AFPFM.

**Figure 7:**
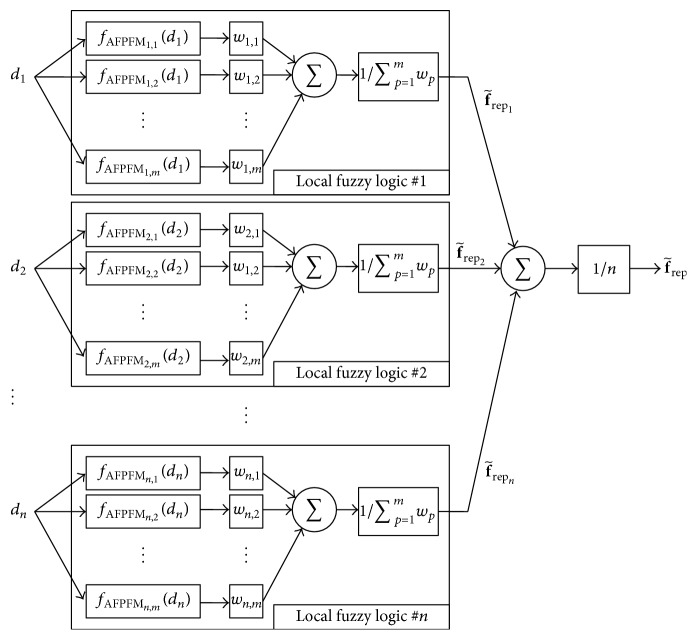
Block diagram of the advanced fuzzy controller with distributed structure.

Figure 7 shows a block diagram of the advanced fuzzy controller for obstacle avoidance, which is designed with a distributed structure. This distributed structure fuzzy controller consists of *n* local fuzzy logic systems, with the *p*th local fuzzy logic yielding intermediate output ${\tilde{f}}_{rep_{p}}$>. In addition, the total output of the distributed fuzzy controller represents the summarized and normalized outputs from all local fuzzy logic systems. Compared to the conventional fuzzy controller that demands *n* × *m* ^*n*^ fuzzy rules for the complete construction of Figure 6, the distributed structure of the fuzzy controller significantly reduces the number of fuzzy rules, and the distributed fuzzy controller for the AFPFM only requires *n* × *m* fuzzy rules. The advanced fuzzy rules of the distributed fuzzy controller can be written as

(22)  p,qth  Rule  Rp,q: IF  dp  is  μdp,q THEN  yp,q=fAFPFMp,qdp,

where *w*_*p*,*q*_ = *μ*_*d*_*p*_,*q*_(*d*_1_), *p* = 1,…, *n*, *q* = 1,…, *m*, and *f*_AFPFM_*p*,*q*__(*d*_*p*_) is the nonlinear output function of the consequent, which can be denoted by

(23) fAFPFMp,qdp=∑i=1nε+didmaxdmaxε+dpkrepp,q1dp−1dmaxsp.

In contrast to the fuzzy rules of (19), the fuzzy rules of (22) have *n* × *m* nonlinear functions in the consequent. As the number of rules in the consequent increases, the repulsive force is more accurately described.

This distributed structure of the fuzzy controller is based on one assumption: all sensors of the robot are independent of each other. According to the independent relationships of the sensors, the distributed fuzzy controller is free from the difficulty of the system design. In contrast to the hierarchical fuzzy controller design, which cannot be based on physical meaning, the structure of this distributed fuzzy controller can be easily identified. The distributed fuzzy controller of the mobile robot equipped with *n* range sensors is decomposed into *n* fuzzy logic systems, as shown in Figure 7, and all local fuzzy logic is designed to represent the subrepulsive forces from their respective sensors. Because the relationships between inputs *d*_1_,…, *d*_*n*_ and intermediate outputs ${\tilde{f}}_{rep_{1}},\ldots ,{\tilde{f}}_{rep_{n}}$> are physically clear and simple, these distributed local fuzzy logic systems can be designed and optimized easily.

The second step in the improvement of the FPFM is the addition of control inputs. As stated above, the FPFM can be improved by completing the fuzzy rules, and the complete fuzzy rules can fully describe the control strategies of experts. Similarly, the additional control inputs increase the number of fuzzy rules, and the additional fuzzy rules describe complex and elaborate control strategies used by the experts. From (15)–(18), the magnitude of the subrepulsive forces is only with respect to the distances between the robot and the obstacles in PFM and FPFM. The AFPFM, however, considers not only the distance between the robot and the obstacles but also additional information involving the relationships among the robot, obstacle, and goal. With this additional information, the fuzzy rules of the FPFM can be enhanced and elaborated, resulting in the more advanced AFPFM.

**Figure 8:**
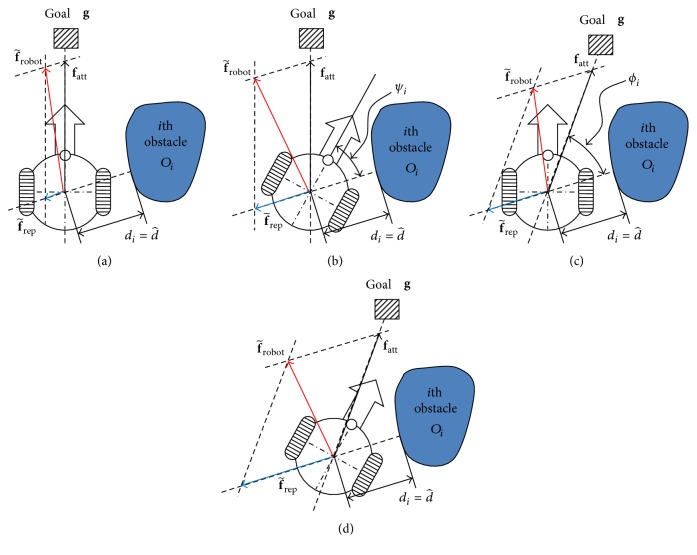
Artificial forces based on the relations among the robot, obstacle, and target.

Figure 8 illustrates the control strategies based on the relations among the robot, obstacle, and goal. The robot moves toward the goal, and there exists one surrounding obstacle. In Figure 8, the repulsive force ${\tilde{f}}_{rep}$> is the total repulsive force of all surrounding obstacles without loss of generality. The magnitude of ${\tilde{f}}_{rep}$> is variable even if the distance *d*_*i*_ between the robot and obstacle is fixed at $\hat{d}$>, in contrast to that in the PFM and FPFM. As shown in Figure 8, the artificial force ${\tilde{f}}_{robot}$> is the force that controls the robot by the magnitude of the repulsive force ${\tilde{f}}_{rep}$>.

Figure 8 shows that the angles *ψ*_*i*_ in Figures 8(b) and 8(d) are small, as are those *ϕ*_*i*_ in Figures 8(c) and 8(d). Thus, the magnitude of the repulsive force ${\tilde{f}}_{rep}$> in Figures 8(b) and 8(c) must be larger than that in Figure 8(a) and smaller than that in Figure 8(d). The additional control strategies to be described by the fuzzy rules of the AFPFM are as follows:

(1) If the robot is heading toward the obstacle (i.e., the angle *ψ*_*i*_ between vectors **s**_*i*_ and **v**_robot_ is small), the magnitude of the repulsive force ${\tilde{f}}_{rep}$> must be large.
(2) If the attractive force **f**_att_ of the robot is heading toward the obstacle (i.e., the angle *ϕ*_*i*_ between vectors **s**_*i*_ and **f**_att_ is small), the magnitude of the repulsive force ${\tilde{f}}_{rep}$> must be large.

**Figure 9:**
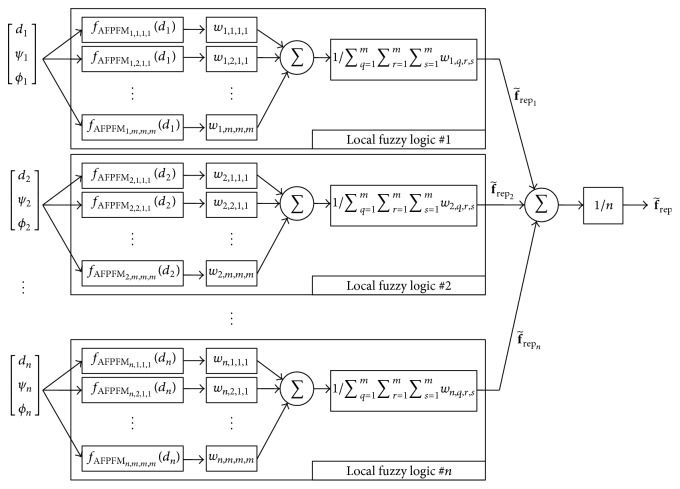
Block diagram of the advanced fuzzy controller with distributed structure and additional control inputs.

Based on these control strategies and the design scheme of the distributed fuzzy controller (refer to Figure 7), the structure of the fuzzy controller for the AFPFM can be designed as shown in Figure 9, and the advanced fuzzy rules for the AFPFM are designed as follows:

(24)  p,q,r,sth  Rule  Rp,q,r,s: IF  dp  is  μdp,q,  ψp  is  μψp,r,  ϕp  is  μϕp,s THEN  yp,q,r,s=fAFPFMp,q,r,sdp,

where *p* = 1,…, *n* and *q*, *r*, *s* = 1,…, *m*. Furthermore, the repulsive force ${\tilde{f}}_{rep}$> of these advanced fuzzy rules can be written as follows:

(25) f~rep=−∑p=1n∑q=1m∑r=1m∑s=1mwp,q,r,syp,q,r,s/∑q=1m∑r=1m∑s=1mwp,q,r,sn,

where

(26) fAFPFMp,q,r,sdp=k~repp,q,r,s∑i=1nε+didmaxdmaxε+dp1dp−1dmaxsp,wp,q,r,s=μdp,qdp∧μψp,rψp∧μϕp,sϕp.

The magnitude of the repulsive forces can be handled by the positive parameter ${\tilde{k}}_{rep_{p,q,r,s}}$>, and these repulsive forces with various scale factors ${\tilde{k}}_{rep_{p,q,r,s}}$> can significantly improve the operational performance of the PFM and the FPFM.

## 3. Simulation

In this section, the performance of the proposed AFPFM for obstacle avoidance is verified by means of simulations using the mobile robot. In the simulations, the performance of the proposed obstacle avoidance method and that of the conventional PFM are compared.

**Figure 10:**
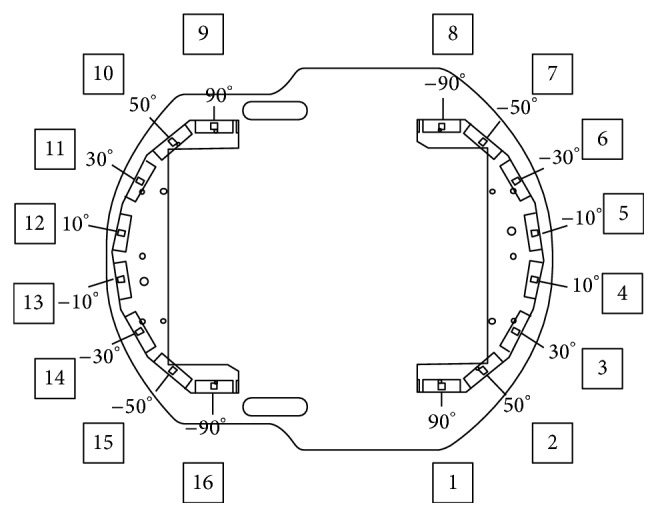
The model of the target control mobile robot and sensor arrangement.

**Figure 11:**
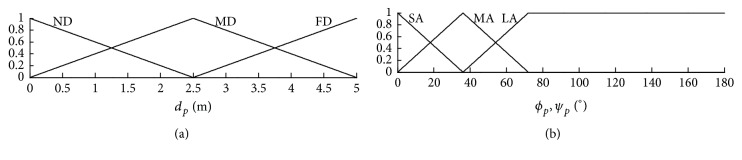
The membership functions of the applied fuzzy controller.

The simulations were executed with Matlab 2009a on an Intel® Core*™*2 Duo CPU E8500 @ 3.16 GHz, 2.00 GB RAM PC. With reference to a commonly used research mobile robot, MobileRobots's Pioneer P3-DX [21], the target control robot was approximated as a circle with a diameter of 0.45 m and could reach speeds of 1.6 m/s. Furthermore, the robot was equipped with 16 sonar sensors, as illustrated in Figure 10. The measurement range of the sonar sensors was from 0.10 to 5 m, and their sampling time was 40 ms. The membership functions of the fuzzy controller are as shown in Figure 11, and the resultant fuzzy rules are shown in Table 1. The primary fuzzy sets denote linguistic meanings such as ND (near distance), MD (medium distance), FD (far distance), SA (small angle), MA (medium angle), and LA (large angle).

The repulsive force scale constant *k*_rep_ of the conventional PFM was determined to be 0.164 by GA [26].

### 3.1. Passing between Closely Placed Obstacles

**Figure 12:**
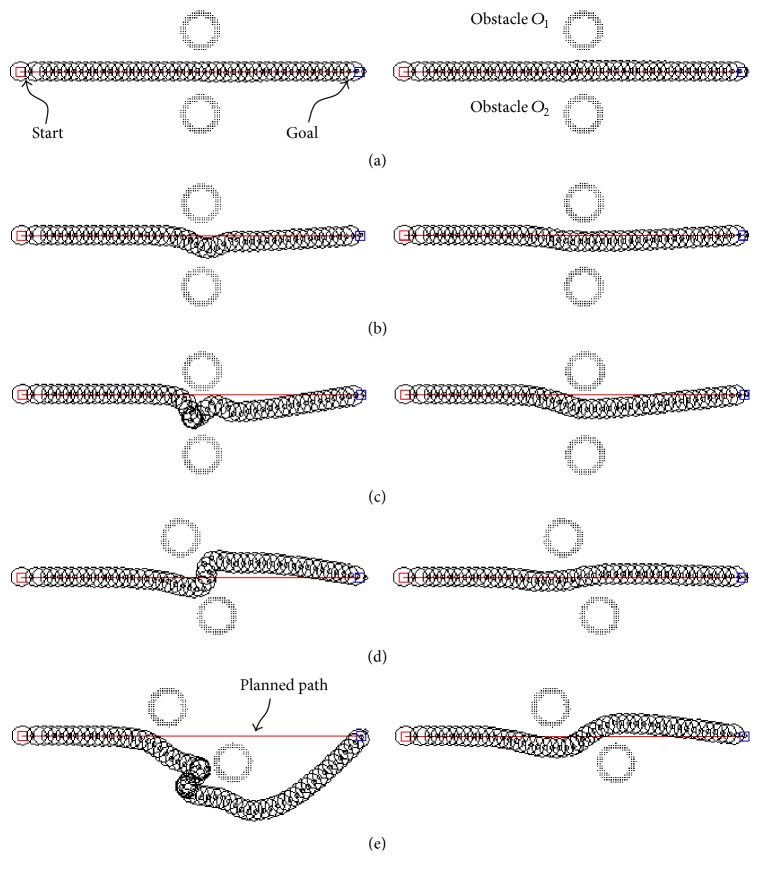
The simulation for passing between closely placed obstacles. Left side: using the conventional PFM; right side: using the AFPFM.

Figure 12 shows the trajectories of the mobile robot when it attempted to pass between two closely placed obstacles: *O*_1_ and *O*_2_. The left- and right-hand sides of Figure 12 show the trajectories of the mobile robot with the conventional PFM and the AFPFM, respectively. The two obstacles were placed at variable positions and orientations while keeping a regular interval (i.e., 1.0 m). Compared to the obstacles shown in Figure 12(a), the topmost obstacles shown in Figures 12(b) and 12(c) were placed 0.3 m and 0.6 m lower, respectively, and those shown in Figures 12(d) and 12(e) were oriented at angles of 25° and 50°, respectively. As illustrated on the left-hand side of Figure 12, the robot that used the conventional PFM was not able to easily and efficiently pass between the two closely placed obstacles. In fact, the trajectories of the robots using the conventional PFM and AFPFM were only similar when the robots approached both obstacles at almost equidistance. In other cases, the robot using the conventional PFM had a sharply curved trajectory. In contrast, the robot using the AFPFM not only passed through the opening between the closely placed obstacles but also had a smooth and short trajectory toward the goal. This minimized the time taken to reach the goal, and the robot did not collide with surrounding obstacles.

The right-hand side of Figure 12 depicts the trajectories of the robot that used the AFPFM. Two obstacles were placed along the path that the robot was to take toward the goal. When the obstacles were placed near a straight line from the robot to the goal, the magnitude of the repulsive force was large on early detection of the obstacles, regardless of the distance from the obstacles. Thus, the robot turned heading direction continuously and slightly in advance of the sharp turn. Because of this predictive avoidance of the obstacle, the trajectory of the robot was smooth and gentle. In addition, the robot generated not only smooth trajectories, but also sharp trajectories when the occasion demanded it, as illustrated by the right-hand side of Figure 12(e). The sharp turn by the robot can only be generated when an obstacle suddenly appears in front of it. After the first slight turn caused by obstacle *O*_1_, the robot was confronted with the second obstacle, *O*_2_. Because the robot was headed in the direction of obstacle *O*_2_ and the distance between them was short, the robot turned sharply to avoid colliding with obstacle *O*_2_ and thus passed between the two obstacles safely. As a result, the robot using the AFPFM avoided the surrounding obstacles efficiently and safely using the appropriate moving trajectories. These appropriate trajectories, depending on the occasion, could be generated by the full and fertile control strategies of experts, which were described by the fuzzy rules of the fuzzy control logic in this study.

### 3.2. Passing through Narrow Passages

**Figure 13:**
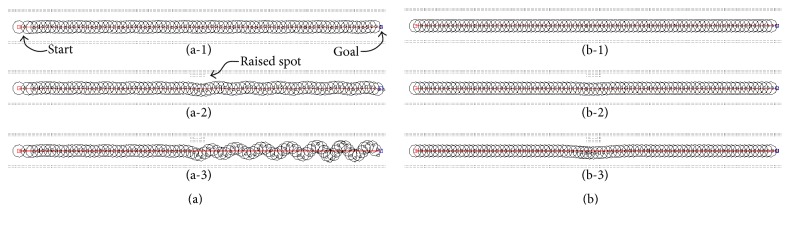
The simulation for passing through the narrow passages: (a) the conventional PFM and (b) the AFPFM.

The simulation shown in Figure 13 represents the robot that passed through narrow passages with raised spots of 0 m, 0.2 m, and 0.4 m in height. Typically, when a robot passes through such passages, raised spots can disturb the robot and cause oscillations. The aim of this simulation, however, was for the robot to exit this obstacle course safely, without wild oscillations. The robot in Figure 13(a) used the conventional PFM, while the robot in Figure 13(b) used the proposed AFPFM. In narrow passages, the robots using the conventional PFM were more sensitive to disturbance and less stable than those using the proposed AFPFM.

Figure 13(a) shows the robot using the conventional PFM passing through the narrow passages. The robot in Figure 13(a-1) passed through the passage that had no raised spots and reached the goal safely, without wild oscillations. However, the robot in Figure 13(a-2) generated oscillating trajectories after passing the raised spots in the passage. Even the disturbance from a small raised spot caused wild, continuous oscillations. In addition, the higher the raised spots, the wilder the trajectory of the robot. This phenomenon is the critical limitation of the conventional PFM, which AFPFM was able to overcome. Figure 13(b) shows the trajectories of the robot that used the AFPFM. In contrast to the robots using the conventional PFM, the robot using the AFPFM passed through the narrow passages without any oscillations. After avoiding the raised spots, the robot returned to the center of the passage smoothly.

## 4. Conclusion

Despite all its advantages, the PFM cannot be used in this context because of its inherent limitations. In this study, the performance of the PFM was improved and incorporated into the design of a proposed AFPFM. As a result, the limitations of the conventional PFM were successfully overcome. Another serious drawback of the conventional PFM is the local minima problem, which has proved elusive for most obstacle avoidance methods including the VFH, VFH+, and VFH*∗*. We plan to combine the AFPFM introduced in this paper with the global path planning algorithm, thereby effectively resolving the local minima problem.

## Figures and Tables

**Table 1:** The resultant fuzzy rules.

(*p*, *q*, *r*, *s*)th fuzzy rule	IF			THEN
	*μ*_*d*_*p*_,*q*_	*μ*_*ψ*_*p*_,*r*_	*μ*_*ϕ*_*p*_,*s*_	${\tilde{k}}_{rep_{i,j,p}}$
*f*_AFPFM_*p*,*q*,*r*,*s*__(*d*_*p*_)	ND	SA	SA	15.000
			MA	3.000
			LA	0.150
		MA	SA	3.000
			MA	0.600
			LA	0.030
		LA	SA	1.050
			MA	0.210
			LA	0.011
	MD	SA	SA	4.500
			MA	0.900
			LA	0.045
		MA	SA	0.900
			MA	0.180
			LA	0.009
		LA	SA	0.315
			MA	0.063
			LA	0.003
	FD	SA	SA	0.150
			MA	0.030
			LA	0.002
		MA	SA	0.030
			MA	0.006
			LA	0.000
		LA	SA	0.011
			MA	0.002
			LA	0.000

Linguistic meanings: ND, near distance; MD, medium distance; FD, far distance; SA, small angle; MA, medium angle; LA, large angle.
